# Why Do We Feel Sick When Infected—Can Altruism Play a Role?

**DOI:** 10.1371/journal.pbio.1002276

**Published:** 2015-10-16

**Authors:** Keren Shakhar, Guy Shakhar

**Affiliations:** 1 Department of Psychology, College of Management Academic Studies, Rishon LeZion, Israel; 2 Department of Immunology, Weizmann Institute of Science, Rehovot, Israel

## Abstract

When we contract an infection, we typically feel sick and behave accordingly. Symptoms of sickness behavior (SB) include anorexia, hypersomnia, depression, and reduced social interactions. SB affects species spanning from arthropods to vertebrates, is triggered nonspecifically by viruses, bacteria, and parasites, and is orchestrated by a complex network of cytokines and neuroendocrine pathways; clearly, it has been naturally selected. Nonetheless, SB seems evolutionarily costly: it promotes starvation and predation and reduces reproductive opportunities. How could SB persist? Former explanations focused on individual fitness, invoking improved resistance to pathogens. Could prevention of disease transmission, propagating in populations through kin selection, also contribute to SB?

## Abstract 2

It remains unclear why infection leads to adverse cognitive, emotional, and behavioral changes. This article introduces the "Eyam hypothesis," suggesting that selection may favor such sickness behaviors because they limit disease transmission to kin.

## Sickness Syndrome and Sickness Behavior

Sickness syndrome is the generalized response of the host to infections. Its classical physiological signs include fever and anemia, but it also includes psychological symptoms—collectively termed “sickness behavior” (SB) [1–3]. These symptoms, familiar to anyone who has been sick, include fatigue, depression, irritability, discomfort, pain, nausea, and loss of interest in food, drink, social interactions, and sex. In animals, such changes can be quantified based on behavior and reflect reprioritization of motivations during disease [2].

**Fig 1:**
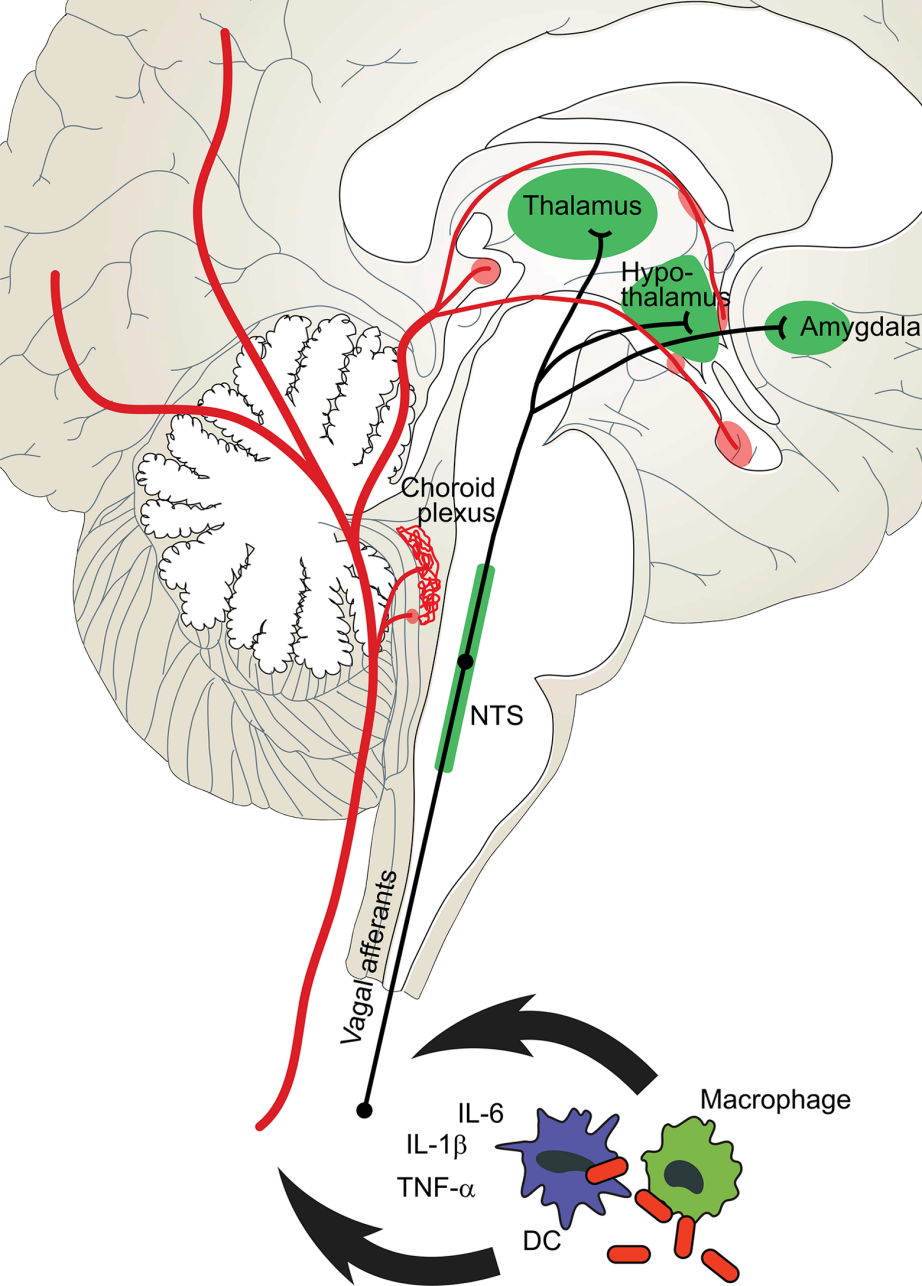
Information regarding inflammation is communicated to the brain through parallel neural and circulatory routes [4,5]. Leukocytes, such as dendritic cells (DCs) and macrophages, sense microbes through pathogen-recognition receptors such as toll-like receptors (TLRs) and NOD-like receptors (NLRs) and then release inflammatory cytokines such as interleukin–1 beta (IL–1β), IL–6, and tumor necrosis factor alpha (TNF-α). In the neural route, cytokines trigger activity in vagal afferents that innervate nuclei in the brain stem such at the nucleus of the solitary tract (NTS). These in turn relay the signal to various nuclei in the hypothalamus, thalamus, and amygdala [4]. In the circulatory route, microbial ligands and cytokines travel through the blood to reach the meninges, choroid plexus, and circumventricular organs (pink) where they can enter the brain. More recent data indicate that such ligands can also activate the epithelium in areas with an intact blood–brain barrier, causing it to synthetize various prostaglandins and release them into nuclei involved in specific behaviors [6].

A common misconception is that pathogens directly produce these behavioral symptoms, but in fact SB is orchestrated by the host’s immune and neuroendocrine systems; mammals have evolved several parallel pathways to alert the brain of inflammation and trigger symptomatic behaviors (Fig 1) [4,5].

Although the specificities may vary, SB is widespread with respect to both pathogens and hosts: diverse pathogens, including viruses, bacteria, and protozoa [1], can trigger it, and equivalent behavioral responses characterize several vertebrate classes [1,2,7] as well as arthropods [8,9]. However, when closely examined, some genera exhibit significant variation in the extent of SB [10], which to date remains unexplained.

## The Mystery—Why Do We Feel Sick?

Since SB is a conserved phenomenon that is mediated by complex immunological and neuroendocrine pathways, it clearly must have evolutionary benefits. Still, in the last 25 years, much effort has been directed at understanding the proximate reasons for SB [3], but its ultimate causation—the reasons SB has evolved in the first place—attracted relatively little attention.

**Fig 2:**
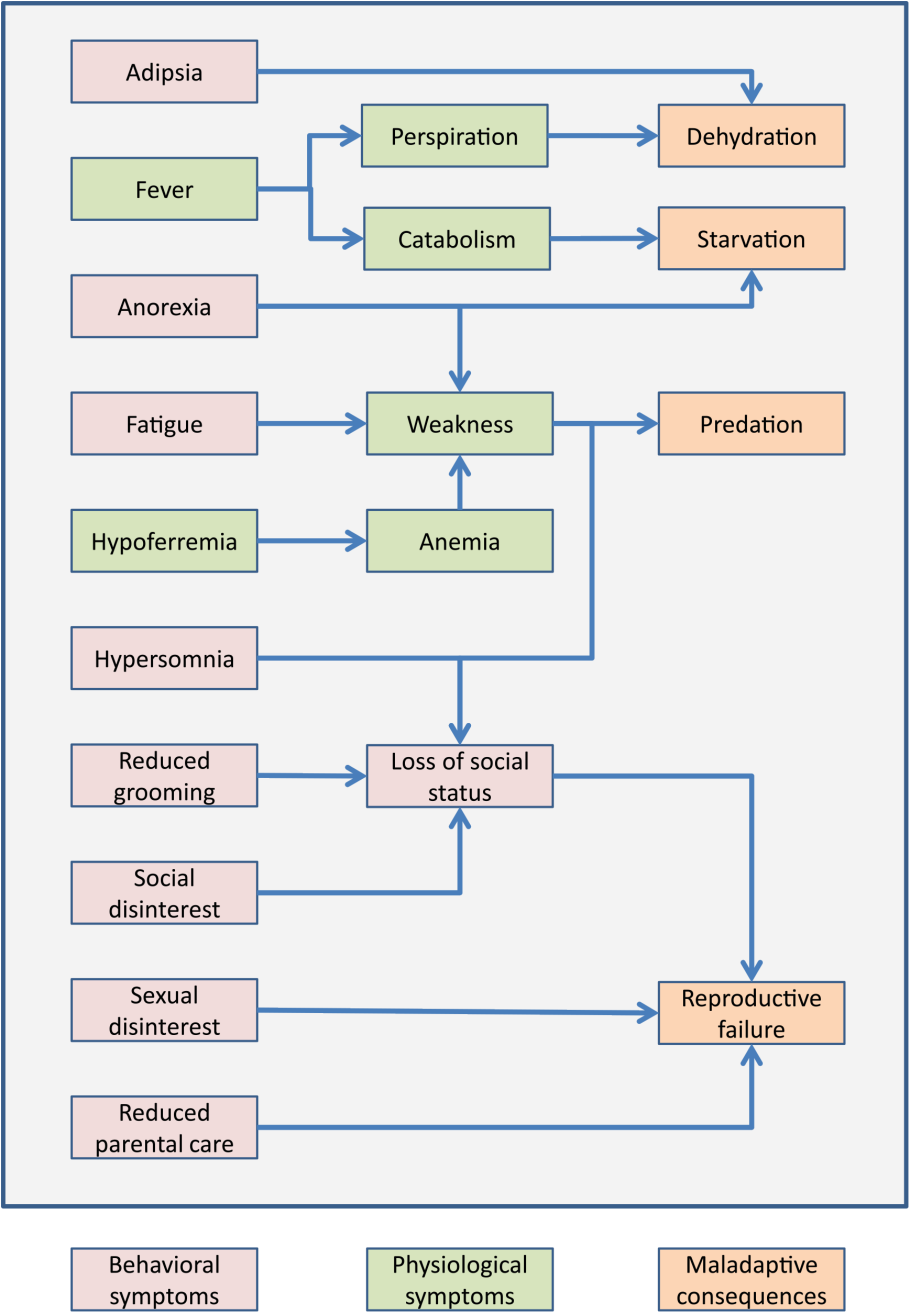
The costs of SB to direct fitness. Behavioral (pink) and physiological (green) symptoms of SB can, either directly or indirectly, lead to maladaptive consequences (orange) that reduce individual fitness.

Unlike physiological symptoms of sickness, such as fever and hypoferremia, which likely boost resistance to pathogens (Box 1), behavioral symptoms remain poorly explained. Clearly, all of these symptoms impose significant costs to host fitness (Fig 2) [11,12]. Anorexia and adipsia increase the risk of starvation, loss of essential nutrients, and dehydration, particularly in the context of fever. Lethargy can lead to predation by slowing down prey and singling it out for predators [13,14]. Social disinterest decreases parental care [15,16], limits mating opportunities [17], and, together with fatigue, can lead to loss of territory and social status [7,18]. For SB to evolve, these costs must be offset by benefits—what can these benefits be?

**Box 1. Fever and Hypoferremia: Physiological Manifestations of Sickness Syndrome**

Physiological responses to sickness are initiated by the immune system and propagated mainly by the brain and liver. Many of these are believed to benefit host resistance to infections, and two, fever and anemia, have been linked to SB [1].

Fever is widely believed to improve survival following infection [19,20] by directly inhibiting the growth of various pathogens and by enhancing immune function (e.g., bacterial clearance, T cell proliferation, and neutrophil activation) [21]. The benefits of hyperthermia has been most convincingly demonstrated in ectoderms such as reptiles and fish, in which deliberate exposure to higher environmental temperatures improved survival [19]. Correspondingly, in rabbits, mice, and chicks, antipyretic drugs repeatedly increased mortality rates from bacterial [22] and viral [23] infections. The evidence in humans is less conclusive, as large-scale blinded trials have not been performed [24,25]. Nonetheless, several small randomized trials have reported that antipyrogenic agents delayed recovery from infections such as malaria [26–28] and chicken pox [29]. Consequently, it has been estimated that routinely treating influenza patients with antipyretics causes at least 700 extra deaths annually in the United States alone [20].

Another physiological component of sickness syndrome is anemia, which is a byproduct of “hypoferremia of infection.” Hypoferremia is a well-regulated process intended to deprive pathogens of the iron essential for their growth [30,31]. It affects several classes of pathogens, including many bacteria, some viruses, and several protozoa. Freely available iron can diminish normal resistance to bacteria in several diseases, and iron overload increased infection rates of pathogens such as tuberculosis, malaria, and brucellosis [31,32]

Infection elicits hypoferremia as part of the hepatic acute phase response [31]. Inflammatory cytokines such as IL–6, IL–22, and type-I interferons trigger the production of the peptide hormone hepcidin in the liver. Hepcidin then binds and internalizes the iron exporter protein ferroportin. As a result, macrophages trap the iron recycled from erythrocytes, and enterocytes stop transferring dietary iron to the circulation, rapidly reducing plasma iron.

## Can SB Improve Host Resistance?

The concept that SB is a coordinated and adaptive response to infections has been established since the mid-1980s. Several comprehensive reviews have covered the historical development of this concept and considered various hypotheses regarding the adaptive role of SB [2–4,14,33].

Early findings suggesting that SB directly benefits the host examined anorexia. In a well-controlled study from 1979, Murray and Murray infected mice with *Listeria monocytogens* and force-fed them to compensate for the resultant anorexia [34]. The treated mice succumbed to the infection at high rates. Unlike the adaptive effects of fever, this remained a largely isolated study, and contesting theories still debate whether anorexia boosts resistance to pathogens and how it might do so [35]. Suggestions included deliberate restriction of nutritional elements, avoiding potentially contaminated food, and a decrease in risky foraging while weak [35]. Newly established routes linking nutrition, intestinal microbiota, and immunity [36] can now also be considered.

In 1988, a seminal paper by Benjamin L. Hart was the first to suggest that SB in its entirety is a coordinated response benefitting the host [1]. Realizing that fever and hypoferremia directly promote host defense (Box 1), Hart suggested that SB is primarily intended to serve these physiological adaptations. Specifically, he proposed that SB evolved to conserve energy needed to sustain metabolically demanding fever. Thus, immobility, lethargy, and reduced motivation to obtain food and drink could have developed to minimize muscle work and exposure to the cold. Anorexia, on the other hand, would promote hypoferremia by reducing iron intake. Other behaviors were viewed as subordinate to the primary ones that conserve energy and reduce iron. Reduced grooming, for example, could preserve fluids in the context of adipsia, whereas decreased foraging would protect a weak animal from predators.

Hart’s hypothesis remained the dominant theory in the field [2,37–40], as it parsimoniously explains a large range of symptoms. Since it was proposed, though, accumulated evidence has exposed some gaps in the hypothesis; it is now time to reassess it.

Conserving energy to maintain fever is central to Hart’s hypothesis. SB is definitely associated with reduced motivation for action—and therefore with less energy expenditure. However, in many cases, fever and SB are decoupled, the one arising without the other [10]. In humans, for instance, malaise and fatigue often characterize mild infections that do not elicit fever. More importantly, several aspects of SB can actually tip the energy balance in the wrong direction. Confinement to nests and dens does not always conserve heat. In warmer climates, dens are cooler than the outside environment and mobility increases body temperature, yet desert animals still remain inside [10]. Another counterproductive symptom is reduced grooming. When mammals and birds stop grooming, their fur and plumage gradually lose their insulating efficiency, requiring more energy to maintain fever [41,42].

The most counterintuitive symptom is anorexia, which, as Hart acknowledged, deprives sick animals of calories needed to fuel fever (especially in migratory animals that cannot reduce energy expenditure by retiring to protected environments). Recognizing this caveat, Hart suggested instead that anorexia evolved to reduce iron consumption, consequently assisting another important antimicrobial response—hypoferremia. It seems unlikely, though, that evolution would favor an indiscriminate reduction in food intake just to decrease iron consumption. Herbivores, for instance, can vary their diet to suit nutritional needs [43], so they could instead avoid only iron-rich foods or ingest clayey soil to interfere with iron absorption [44].

More importantly, physiologists have since gained much mechanistic insight into hypoferremia, rendering this notion less likely. Dietary iron absorption is dwarfed by the total iron reserves in the human body and the amount recycled through erythropoiesis [45]. Anorexia, therefore, can only mediate slow-acting changes in plasma iron [46]. In contrast, inflammatory agents such as lipopolysaccharide (LPS) can halve plasma iron within a few hours [47]. The direct mechanism through which infection elicits hypoferremia (Box 1) was only discovered 15 years ago [31] and involves the rapid production of hepcidin in the liver. This efficient mechanism obviates anorexia when infection requires the host to rapidly reduce plasma iron.

Sensing that Hart’s explanation cannot account for all the symptoms of SB, several complementary theories have since been proposed. Watkins and Maier [48] stressed the importance of allodynia and hyperalgesia (reduced threshold and increased intensity of pain) in SB. They proposed that these symptoms, together with the reduced activity SB introduces, are intended to protect sensitive organs and tissues from further damage. Medzitov et al. [49] maintained that SB chiefly promotes tolerance towards parasites, rather than their clearance, although the details of this interaction remained unclear. All these theories focus on direct benefits that infected individuals may derive from SB; they disregard the indirect effects SB may have at the group level.

Overall, the evidence that all the symptoms of SB directly improve host resistance to infection remains incomplete, and after several decades of research in this field, writers still debate whether and how symptoms of SB benefit hosts [3,14,33,35,50]. What, then, could a complementary evolutionary explanation be?

## Could Kin Selection Drive the Evolution of SB?

If gains to direct fitness cannot fully explain SB, perhaps inclusive fitness could come into play. We propose that reduced transmission of infectious disease among related individuals contributed to the evolution of SB. Although the idea that SB reduces transmission has been alluded to before [3,20,51,52], it was never recognized as a major organizing principle for SB in vertebrates. We name this theory “the Eyam hypothesis” after the English mining community that isolated itself to contain an outbreak of bubonic plague in 1666. Three-quarters of the villagers reportedly died, but the surrounding communities were saved [53].

The Eyam hypothesis relies on three premises:

## Premise 1: SB Reduces Direct and Indirect Contacts between Infected Individuals and Their Conspecifics

**Fig 3:**
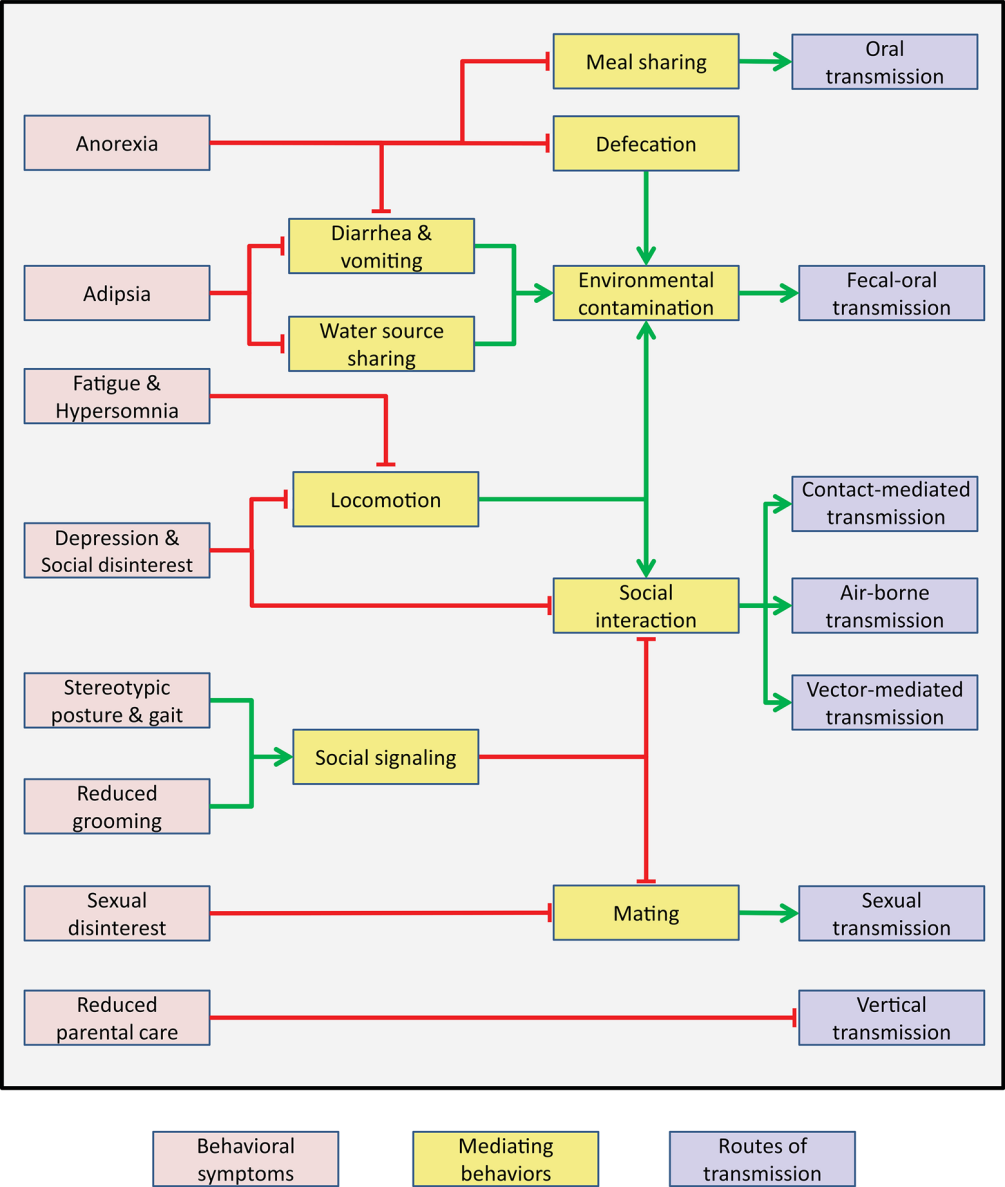
The benefits of SB to indirect fitness. Symptoms of SB (pink) can suppress (red connectors) or promote (green arrows) several mediating behaviors (yellow), consequently reducing pathogen transmission through several routes (blue).

Strikingly, most of the symptoms that constitute SB share a common denominator: they restrict contacts between sick individuals and their social groups (Fig 3). Symptoms of sickness achieve this feat using three containment strategies:

### Containment Strategy #1: Restricting Physical Contacts

It is self-evident that salient symptoms of SB, such as social disinterest, depression, hyperalgesia, fatigue, and hypersomnia, reduce the mobility and social activity of infected individuals, limiting their contact with conspecifics. Likewise, sexual disinterest suppresses courtship and mating behaviors, whereas reduced parental care entails by definition less interaction with offspring. The contribution of anorexia and adipsia may be less apparent; by suppressing the motivation to eat and drink, they reduce the urge to travel in search of food and water, share meals with group members, and gather at water sources.

Self-imposed isolation may account for the folk observation that terminally ill dogs leave their owners to die alone. Similar behavior has been recorded in the wild among badgers, which, when infected with bovine tuberculosis, separated from their clan and settled in individual setts, where they died [54].

Tellingly, the opposite effect is observed when pathogens manipulate host behavior to their benefit. In such diseases, infected hosts become hyperactive and interact more with potential hosts: for example, rabid dogs become fearless and bite, and rodents infected with *Toxoplasma gondii* lose their fear of cats (the definitive hosts) [55].

### Containment Strategy #2: Limiting Environmental Contamination

On top of restricting direct contacts, SB can also limit indirect contacts between conspecifics by reducing microbial contamination of shared resources: ground, food and water (Fig 3).

Symptoms such as hypersomnia, fatigue, and depression restrict the animal’s radius of activity, limiting environmental contamination to its immediate surroundings. Social and sexual disinterest, as well as anorexia and adipsia, further reduce the drive of animals to travel farther afield.

Anorexia and adipsia seem paramount in that respect as they prevent sick animals from contaminating shared food and water resources. Contamination of pastures (for herbivores) or carcasses (for carnivores) and contamination of water holes are undoubtedly major routes for oral and fecal-to-oral transmission in the wild. Finally, anorexia and adipsia also reduce defecation, diarrhea, and vomiting, which are the major means of spreading for enteric pathogens.

### Containment Strategy #3: Advertising Infection to Conspecifics

Whereas strategies #1 and #2 involve self-imposed restrictions, SB can also act by provoking responses from conspecifics. In many species, group members can detect infected individuals through visual, olfactory, and chemical cues [56–59], distance themselves, and stop interacting with them [60]. Such signaling has been demonstrated most convincingly in eusocial insects in which chemical communication is used to coordinate social immunity (Box 2).

**Box 2. The Case for Social Immunity in Eusocial Insects**

Eusocial insects—social bees and wasps, ants, and termites—form colonies dubbed “superorganisms.” These contain few breeding individuals and many closely related sterile workers. Workers are dispensable, care collectively for brood, and are genetically investing in their siblings and parents. This situation encourages cooperation and altruism. Colonies of eusocial insects are ideal settings for the spread of pathogens, as their inhabitants live at high density, constantly touch one another, and exchange food orally. Low genetic diversity may pose an additional risk, as more individuals are susceptible to the same pathogens. Theoretically, these factors make eusocial insects optimal candidates to develop SB.

Empirically, it has long been recognized that eusocial insects exhibit social immunity, collective behaviors that promote parasite resistance [61] and limit contagious interactions among group members [62]. Many of these behaviors resemble SB in vertebrates, whereas some are idiosyncratic adaptations to the situation in insect colonies.

Specifically, among several species of ants, individuals that had been experimentally treated with live pathogens or pathogen-associated molecules (such as LPS) are less sociable [8], avoid contacting brood [8,63], stop transferring food to nest mates (trophallaxis) [9], become less motile [9], decrease allogrooming of nest mates [64], and spend most of their time outside the nest, where they eventually die [8,65]. Similarly, among honeybees, individuals whose health is compromised eat less, transfer less nectar to the hive [66], spend less time in the hive [67], and leave it to die in isolation [66]. This compulsion to leave the hive may explain sudden mass desertions observed in the recent epidemic of collapsed colony disease (CCD), regardless of the elusive pathogen that induces it [68]. The behavior of parasitized termites has been less studied, but infected individuals seem to migrate to bottom strata of mounds and die there [69].

Communicating health status is an important aspect of social immunity. The bulk of our knowledge concerns “hygienic behavior” in honeybees. In this process, infected larvae and pupae are detected and removed from the hive by workers, limiting the spread of infections [70,71]. Evidently, the brood communicates its health status chemically at the earliest sign of infection. Recently, it was shown that adult bees can also be expelled from the hive based on similar signals [71]. The behavioral component of such signaling is clearer in dampwood termites in which adults that have contacted fungal spores signal through vibration to repel colony members [60].

Studies in rodents implicated the vomeronasal organ in sensing infection [72] and discouraging social and sexual interactions [59]; importantly, immune activation with LPS was enough to mark animals as sick. Even in humans, mammals with an ill-reputed sense of smell, the clothes of LPS-treated subjects can be sniffed out [73].

It is easy to accept that the detection of infected conspecifics has evolved as a protective avoidance mechanism, but the transmission of such signals could also have been selected for. Several symptoms of SB may act as infection cues: reduced self-grooming visibly distinguishes infected individuals as scruffy [1] and probably accentuates the olfactory signals they emit. Similar changes may affect vocal communication. In sparrows, for instance, the frequency and pattern of birdsong change during an inflammatory response [74]. Lastly, the stereotypic posture and motion that infected animals adopt because of fatigue and hyperalgesia can act as additional cues. Thus, LPS-treated subjects can be detected by observers based on their gait [75]. The signaling aspects of such behavioral changes are exposed by the response of sick animals to predators. Under the gaze of carnivores, sick members of a herd would attempt to disguise their vulnerability and suppress SB [14]. This observation suggests that animals can alert their kin of infection but suppress such signaling to predators.

## Premise 2: Reduced Contacts Limit the Spread of Infections

Medicine has long acknowledged the importance of isolation for containing infectious disease in humans. Behavioral interventions such as quarantine, school closures, and bans on travel and public gathering have curtailed the spread of contagious diseases such as Ebola [76], vector-mediated diseases such as bubonic plague [77], and airborne ones such as severe acute respiratory syndrome (SARS) [78]. These successes demonstrate that, regardless of the route, social isolation can reduce transmission.

A question more relevant to the evolution of SB is whether self-imposed social isolation is effective in the wild. Several such examples exist: in the last decade, bat populations of many species in North America collapsed because of the “white nose” fungal disease. Although almost all of the colonies observed were decimated, some bat populations survived by adopting a solitary roosting pattern [79]. Conversely, a study in wild deer mice has shown that highly active individuals, which encountered more mice, exhibited higher viral infection rates [80].

Isolation of infected people based on clinical symptoms can be effective only when they overlap with the infectious period [81]. Empirical data suggest that, in the few infectious diseases studied (barring HIV), this is indeed the case. Thus, in SARS, smallpox, and foot-and-mouth disease, this overlap exceeds 80% [81,82], and estimates for influenza range between 50% and 90% [81,83]. Since behavioral symptoms typically precede specific clinical signs, these figures likely underestimate the overlap between SB and infectivity and the potential reduction in transmission.

## Premise 3: Behaviors That Reduce Pathogen Transmission Can Persist through Kin Selection

If indeed SB favors the fitness of other group members at the expense of the individuals, then it can be considered an instance of biological altruism. It has long been debated how altruism can become an evolutionarily stable strategy (ESS). A likely mechanism is kin selection, the positive selection of traits that increase the fitness of the individual’s relatives. This initially controversial theory, put forward by W. D. Hamilton [84], has been mathematically validated and widely accepted since [85].

Kin selection is easy to accept when altruism is actively directed at relatives (e.g., birds feigning injury to lead predators away from their chicks), but how can it promote SB, a response that indiscriminately favors related and unrelated group members? This can only happen when the average relatedness within the social group is higher than within the entire population. Indeed, in many (although certainly not all) species, genetically related individuals are disproportionally represented in the immediate social groups in which most physical interactions occur [86–89]. Such bias develops because of high population viscosity, i.e., slow and spatially restricted dispersal of progeny.

Animal species vary in the degree of intergroup relatedness based on their life history. At one end of the spectrum are r-strategists whose offspring are neonatally independent and disperse widely. Under such conditions, social considerations are unlikely to drive SB. At the opposite end of the spectrum lie eusocial animals. Among these, eusocial hymnoptera have been studied most (Box 2). These insects indeed display a variety of collective disease defense behaviors, in part resembling SB, which are collectively termed “social immunity” [61]. Humans, classical K-strategists who cohabit most of their lives with first-degree relatives, seem to lie closer to this pole.

Intriguingly, some experimental evidence suggests that SB is actually not as universal as commonly assumed. Some birds can become infected, mount an immune response and develop fever without showing conspicuous signs of illness [90], leading to an apparently sudden death from infection [14]. In fish, administration of LPS triggers no observable behavioral changes [91]. Studies in wild mouse populations showed that the intensity of SB varies considerably among related species [10]. How this diversity relates to social structure is yet to be examined.

## Where Do We Go from Here?

The Eyam hypothesis has never been directly tested, so the empirical evidence supporting it is still limited; nonetheless, it produces testable predictions. As stated above, an ESS for SB would counterbalance its cost to the infected individuals with the benefit of reducing transmission to their kin. This benefit should be proportional to pathogen virulence, the chances of transmission between individuals (infectivity), and the average relatedness of susceptible hosts. Consequently, several predictions can be examined either correlatively (1-3), experimentally (4, 5), or mathematically (6, 7).

1. **Virulence:** Different pathogens invoke SB of varying intensities. Our theory predicts that, through an evolutionary process, more virulent pathogens would come to provoke stronger behavioral responses. When a pathogen is deadly, the individual loses little (as it would die anyway) and gains much (as it saves its relatives from death) from a debilitating behavioral response. When a pathogen is avirulent, the optimal behavioral response would be a subclinical one, invoking no SB even if an immune response is activated.
2. **Disease transmission:** Increased odds for transmission would also favor a vigorous SB. Thus, more intense SB is expected among species that live in dense colonies and engage in close physical contact (especially in gregarious seasons). Likewise, highly contagious pathogens are expected to trigger a more pronounced SB.
3. **Genetic relatedness:** Higher relatedness within social groups should also promote SB (as it would other altruistic behaviors). Thus, SB would intensify with population viscosity and the length of care for offspring. This could be tested by comparing phylogenetically close species (e.g., social versus solitary wasps). Comparisons can also be made among individuals within communities: for example, mothers versus fathers in polygamous species and reproductive versus nonreproductive members of eusocial communities.
4. **Anti-inflammatory drugs:** Pharmacologically suppressing SB in experimentally infected individuals should accelerate the spread of infections even when the course of disease is unaltered. Such experiments, though, would require habitats that allow efficient self-isolation.
5. **Tracers:** To rule out immunological effects on pathogen clearance, the dispersal of innocuous tags such as pigments or radioisotopes can be traced.
6. **Mathematical models:** These could be developed to formally examine the feasibility of the hypothesis.
7. **Computer simulations**: These could be applied to test the hypothesis numerically.

We are so used to malaise being the essence of infection that we often forget to ask why it evolved. The social implications of SB may not be the only selective force driving it, but they clearly contribute and have been disregarded for too long. The mystery of SB is not only intellectually provoking but also clinically significant. This is because behavioral symptoms are routinely relieved using anti-inflammatory drugs such as cyclooxygenase (COX) inhibitors. According to the Eyam hypothesis, such use could prove socially irresponsible. By enabling infected people to travel widely and socialize, it interferes with a natural mechanism that prevents pathogen spread. In contrast, SB that accompanies medical procedures (such as cytokine treatment) and noninfectious diseases (such as cachexia in cancer) is a side effect that could be treated safely.

## Abbreviations

CCD: collapsed colony disease
COX: cyclooxygenase
DC: dendritic cell
ESS: evolutionarily stable strategy
IL: interleukin
LPS: lipopolysaccharide
NLR: NOD-like receptor
NTS: nucleus of the solitary tract
SARS: severe acute respiratory syndrome
SB: sickness behavior
TLR: toll-like receptor
TNF-α: tumor necrosis factor alpha
